# The New Orleans Medical and Surgical Journal

**Published:** 1889-01

**Authors:** 


### THE NEW ORLEANS MEDICAL AND SURGICAL JOURNAL.

Because forsooth, we mildly and courteously protested against the wholesale and sweeping denunciation of the members of the Louisiana State Medical Association by this, its fostered organ, in its now notorious June editorial, and suggested that both as a matter of right, justice and good business policy, the Journal should make, or should have made *some exceptions*, we are accused by this publication of "an act derogatory to Southern journalism!" Because we pointed out and insisted that there are honorable, honest, able and conscientious physicians in that body, well known to that journal as such, who cannot, with justice be denounced as "languid, inconsequent and unprepared medicine men," and whose high aim and object in assembling in convention cannot be impeached, much less can these gentlemen—all of them—be accused of being "bent for the most part on a few days of rest, cigar-smoking and story-telling," our *animus* towards that journal is impugned, and we are accused of unfriendliness, and told that it, the said journal, "would blush for very shame" if accused of something similar.

We protest that our article was penned in the kindliest spirit toward our contemporary, and that heretofore, we have had no occasion to entertain other than a friendly feeling for our neighbor; why should we? Personally, we are not acquainted with a single member of its copious staff; they have done us no harm; why should we be unfriendly toward their little enterprise? They are not in our way in the least, and the Lord knows we do not envy them. But, whatever may have been our animus toward them, we claim the right, which it is absurd to deny any journalist, and yet which they would deny us, to comment on matters of medical news or controversy in other states beside our own. We are given to understand that any matters of this kind, even the proceedings of medical societies, are *private property* in a State, and that no journal outside of the limits of that State can discuss them without being "derogatory to Southern journalism." Can anything be more absurd and ridiculous? We presume then, in the eyes of our wrathy contemporary, the N. Y. Medical, Record and all other journals that have discussed the Anglo-German medical controversy, are guilty of an act "derogatory" to German "journalism?" We opine that if our neighbor were to practice what it preaches, and confine itself to the profession of Louisiana alone for its medical news, it would be at a loss for copy occasionally. We observe, however, in its columns considerable Texas news, copied from the Texas journals, with more or less comment.

With regard to "blushing for very shame," (that journal expresses itself as ready to "blush" if accused of an animus toward a contemporary such as it imputes to us toward themselves,) we will say, if it is capable of such an act there is abundant room for its performance whenever is recalled their own animus in be-little-ing and traducing their own State Association. "It is a sorry bird that befouls its own nest."

The only thing for which we feel that *we* should "blush", is our want of discernment and discrimination in so long regarding and styling the *N. O. Medical and Surgical Journal* a "courteous contemporary." But, then, we did not know that there was upon the staff a gentleman of such capabilities as revealed in the rather coarse, and eminently unjust tirade against this JOURNAL, in its December issue.

The *N. O. Medical and Surgical Journal* has simply declared that the horse is sixteen feet high, and *sticks to it*, notwithstanding the abundant and everwhelming proof to the contrary.

Finally, our extracts from the said June editorial are declared "garbled and misleading," They are neither.

This seems to be a sore subject with our neighbor, and we forbear. Perhaps we should not have mentioned it, but having done so, we believe we are right, and will defend ourselves and our position. That "truth hurts," is an old saying; and a man in the wrong does not relish having it pointed out to him, because he feels that he ought to acknowledge it; and there are some men who are not capable of it; who cannot rise to the sublimity oi such a confession, however palpable may be their error.

